# Occurrence and Seasonal Variations of Lipophilic Marine Toxins in Commercial Clam Species along the Coast of Jiangsu, China

**DOI:** 10.3390/toxins8010008

**Published:** 2015-12-25

**Authors:** Xin-Zhi Wang, Ying Cheng, Na Li, Hong-Mei Wen, Rui Liu, Chen-Xiao Shan, Chuan Chai, Hao Wu

**Affiliations:** 1 School of Pharmacy, Nanjing University of Chinese Medicines, Xianlin Avenue No. 138, Nanjing 210023, China; wxzatnj@sina.com (X.-Z.W.); njwenhm@126.com (H.-M.W.); cpulr@126.com (R.L.); thomastiger@163.com (C.-X.S.); echo_0523@hotmail.com (C.C.); 2 Marine Drug Research and Development Center of Jiangsu Province, Xianlin Avenue No. 138, Nanjing 210023, China; chengyingnj@163.com (Y.C.); linna7249@sina.com (N.L.)

**Keywords:** lipophilic marine toxins, UFLC-TQ-MS, clams, seasonal variations, Jiangsu Province

## Abstract

Recent studies have examined lipophilic marine toxins (LMTs) in shellfish and toxic algae worldwide, but the occurrence and seasonal variations of LMTs in commercial clams (including *Mactra veneriformis*, *Ruditapes philippinarum*, *Meretrix meretrix*, and *Cyclina sinensis*) at their major culturing area in Jiangsu, China, remain largely unexplored. In this study, a new solid phase extraction (SPE) in combination with an ultra-fast liquid chromatography and triple-quadrupole linear ion trap mass spectrometry (UFLC-TQ-MS) method was developed to determine the presence of 10 typical LMTs (okadaic acid (OA), yessotoxins (YTXs), azaspiracids (AZA1-3), pectenotoxins (PTX2), gymnodimine (GYM), dinophysistoxins (DTX1&2), and spirolides (SPX1)) in the aforementioned four clam matrices. After confirmation of its sensitivity and precision, this method was used to evaluate the amounts of LMTs in clam samples harvested in five aquaculture zones of the Jiangsu coastal area. Monthly variations of GYM, PTX2, OA, and DTX1&2 in 400 clam samples from the sample areas were determined from January 2014 through August 2015. Peak values were observed during May and August. This is the first systematic report of LMTs detected in clam samples harvested in Jiangsu. Follow-up research and the implementation of protective measures are needed to ensure the safety of clams harvested in this area.

## 1. Introduction

**Figure 1:**
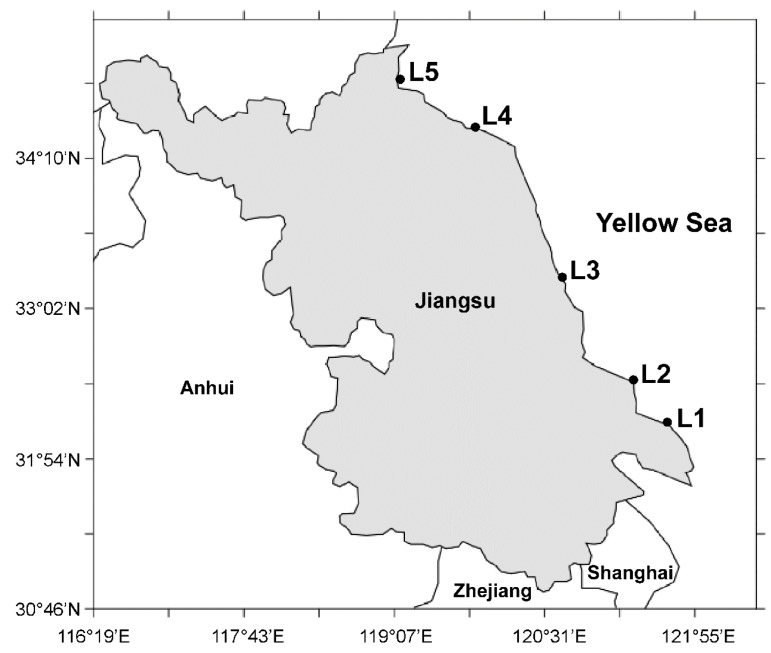
Sample collection locations along the Jiangsu coastline. L1 represents Maojia harbor (121°40ʹ E, 32°01ʹ N), L2 represents Lvsi harbor (121°37ʹ E, 32°05ʹ N), L3 represents Dafeng harbor (120°50ʹ E, 33 °16ʹ N), L4 represents Ganyu harbor (121°49ʹ E, 34°29ʹ N), and L5 represents Lianyun harbor (119°20ʹ E, 34°46ʹ N).

The hard clams *Mactra veneriformis* (surf clam), *Ruditapes philippinarum* (steamer clam), *Meretrix meretrix* (Asiatic hard clam), and *Cyclina sinensis* (Venus clam) are commercially dominant species harvested in the coastal areas of south and southeast China [1]. The coastal tidal flat of Jiangsu Province, with its particularly favorable natural conditions, is a major culturing area for these clams (See Figure 1). Over 628 kilotons of hard clams were harvested from this area in 2012, many of them for export to Japan, Europe, and other regions [2,3,4,5,6,7].

Lipophilic marine toxins (LMTs), including okadaic acid (OA), dinophysistoxins (DTXs), azaspiracids (AZAs), yessotoxins (YTXs), pectenotoxins (PTXs), gymnodimine (GYM), and spirolides (SPXs), are secondary metabolites produced by marine algae (chemical structure see Figure S1) [8,9]. Unfortunately, these toxins can accumulate in the organs and tissues of clams via their feeding behavior [10]. Once consumed by humans, some of the toxins, including OA, DTXs, and AZAs, can cause diarrhea, nausea, vomiting, and abdominal pain [9,10], commonly known as diarrhetic shellfish poisoning (DSP), whereas others, such as YTXs, PTXs, SPXs, and GYM, reportedly test positive for detection by means of the traditional mouse bioassay (MBA) [11,12,13], but intoxication in humans have not been reported.

The phytoplankton responsible for LMTs include *Prorocentrum lima*, a range of *Dinophysis* species, *Gymnodinium*, *Azadinium poporum* and *Karenia brevis* [14,15]. The distribution and frequency of occurrence of LMT-producing microalgae have increased throughout the world in recent decades [8,9,10,14,15,16,17,18,19,20,21,22,23,24,25,26,27,28]. In China, there have been reports of the aforementioned algae species and episodes of LMT accumulation in shellfish along the coasts of Liaoning, Shandong, Zhejiang, Fujian, and Guangdong provinces [29,30,31,32,33,34,35,36]. In 2011, a major incident occurred in coastal cities along the East China Sea. More than 200 individuals received clinical treatment for symptoms including diarrhea, nausea, vomiting, and abdominal pain after consuming mussels that had been shipped from a harvest site in Fujian Province. High concentrations of OA, DTX1, and PTX2 toxins were determined to be the cause of the episode [36]. This event highlights the importance of developing an effective monitoring program for LMTs in seafood in China to protect human health and the aquaculture industry.

In China, the protocol of mouse bioassay (MBA) was widely used to detect LMTs since 1994 [37]. However, the MBA is not specific for LMTs, and other marine toxins may produce positive results. Moreover, some studies claim that the fatty acids in shellfish may induce “false positives” in MBA tests [38]. Therefore, much effort has been invested in the development of instrumental methods that are dedicated to either detecting specific classes of LMTs, or detecting as many different LMTs as possible in a multi-toxin method [39,40,41,42,43,44,45,46]. Until July 2011, the European Union (EU) decided to utilize liquid chromatography with tandem mass spectrometry (LC-MS/MS) as a reference method for LMTs monitoring as it provides a more specific and sensitive technique for quantification of the full range of LMTs [47]. Since then, LC-MS/MS has become an essential research tool for LMT monitoring in China. By using this method, many LMTs including OA, DTX1&2, PTX, GYM and AZAs have been detected for the first time in mussels [29,32,33,34], oysters [32,33,34], scallops [32,33,34], seawater [31] or microalgae [30,35] samples along the coast of China.

Even though the coastline of Jiangsu is one of the major culturing areas of clams in China [1,2,3,4,5,6,7], to the best of our knowledge, no recent research has focused on the occurrence and seasonal variations of LMTs in clam samples cultivated in this region. Moreover, although similar work has been done in other shellfish matrices in China [29,32,33,34], the detection of multiple LMTs in the aforementioned clam matrices using the LC-MS/MS method has not been reported. Therefore, the aims of this study were (1) to establish a rapid and sensitive routine LMT monitoring method using the ultra-fast liquid chromatography-tandem mass spectrometry (UFLC-MS/MS) for clams harvested in Jiangsu; (2) to provide a basic understanding of the current contamination levels of dominant clam species cultivated in different aquaculture zones of Jiangsu by investigating occurrence and monthly variations of each LMT in four species (*M. veneriformis*, *R. philippinarum*, *M. meretrix*, and *C. sinensis*) during the course of January 2014 to August 2015; and (3) to obtain information that will help ensure the safety of the clams cultivated in this area. This is the first time that multiple LMTs have been analyzed by monthly acquired clam samples from aquaculture zones in Jiangsu.

## 2. Results and Discussion

### 2.1. UFLC-MS/MS and Pre-Treatment Method Optimization

#### 2.1.1. Optimization of Chromatographic Separation

Previous studies have optimized chromatographic separation and signal intensity using shellfish extracts spiked with mixed LMT standards. The chromatographic separation of LMTs was primarily performed on C_18_ columns [39,40,41,42,43,44,45,46,47]. The use of a mobile phase containing ammonium hydroxide instead of formic acid or ammonium acetate significantly improved the sensitivity and peak shape of some LMTs [42]. Based on this information, a Waters Xbridge™ C_18_ column was selected for this study because it contains a crossed-linked type of silica, which is stable over alkaline conditions.

**Figure 2:**
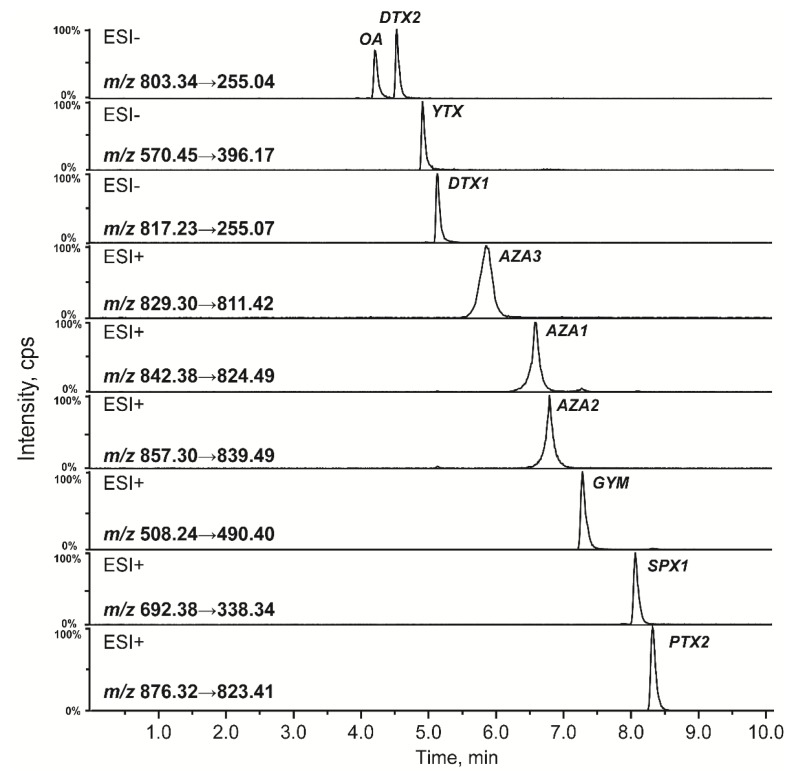
Separation of ten LMT standards using the XBridge™ C_18_ column under alkaline conditions.

The concentration of ammonium hydroxide in the mobile phase was optimized in this study. The ionization efficiency of OA, DTXs, and peak shape of YTX improved significantly with increasing ammonium hydroxide concentrations. However, the matrix effect became stronger with an increase of pH in the mobile phase, and subsequently led to signal suppression of the other analyzed toxins. A 5 mM ammonium hydroxide concentration was ultimately selected to obtain satisfactory peak shapes and MS response of all analyzed LMTs. Representative multi-reaction monitoring (MRM) chromatograms of the LMT standards under optimized condition are shown in Figure 2.

#### 2.1.2. Optimization of MS/MS Parameters

Standard solutions of each analyte were infused into the mass spectrometer to detect ions and optimize mass parameters. OA, DTX1, and DTX2 were preferably analyzed in negative electrospray ionization (ESI^−^)because they had a satisfactory MS response in negative mode, with [M − H]^−^ ions at *m/z* 803.5, 817.6, and 803.5, respectively. The MS response of YTX could only be observed in negative mode, with a double negatively charged [M − 2H]^2−^ ion at *m/z* 570.4. AZA1, AZA2, AZA3, GYM, and SPX1 had a sufficient MS response of [M + H]^+^ at *m/z* 842.8, 857.3, 829.3, 508.5, and 692.3, respectively, whereas PTX2 had an excellent MS response of [M + NH_4_]^+^ at *m/z* 876.3 in positive mode.

Based on the confirmation of the parent ion, two or more daughter ions should be selected when low-resolution LC-MS analysis is used, in accordance with relevant EU legislation [33]. Therefore, the optimization of daughter ions and their declustering potential (DP), collision energy (CE), and collision cell exit potential (CXP) was performed under the compound optimization tool of Analyst 1.5.2 software. The MS/MS transitions for quantification and confirmation, as well as the DP, CE, and CXP values optimized for each of the target compounds, are shown in Table 1.

**Table 1:** Mass spectrometry (MS/MS) conditions used for the multi-reaction monitoring (MRM) acquisition windows for the detection of lipophilic marine toxins (LMTs).

Toxins	ESI Mode	Precursor (*m/z*)	Product (*m/z*)	CE	DP	CXP
GYM	ESI^+^	508.24	490.40	33	206	42
			160.2	55	206	20
PTX2	ESI^+^	876.32	823.41	33	36	34
			213.1	45	36	12
SPX1	ESI^+^	692.38	338.34	25	41	36
			164.2	56	41	32
AZA1	ESI^+^	842.4	824.49	41	81	34
			672.4	68	81	45
AZA2	ESI^+^	857.3	839.49	42	21	34
			673.5	65	21	52
AZA3	ESI^+^	829.3	811.42	39	11	34
			659.4	61	11	39
OA	ESI^−^	803.5	255.04	−62	−30	−17
			112.80	−67	−30	−15
DTX1	ESI^−^	817.2	255.07	−62	−50	−19
			113.04	−54	−50	−13
DTX2	ESI^−^	803.5	255.09	−58	−45	−21
			113.08	−30	−45	−15
YTX	ESI^−^	570.45	396.17	−80	−45	−18
			476.35	−31	−45	−44

#### 2.1.3. Optimization of the Sample Pre-Treatment Method

As noted in previous studies, polymetric sorbent is suitable to clean up the matrix of shellfish samples, and it maintains satisfactorily reproducible recoveries for the enrichment of LMTs [41]. Therefore, in this study, Oasis HLB cartridges (Waters, Dublin, Ireland), Strata-X cartridges (Phenomenex, Torrance, CA, USA), and Bond Elut Plexa cartridges (Agilent, Santa Clara, CA, USA) with polymetric sorbent were compared in terms of extraction of 10 LMTs in spiked clam samples. As shown in Figure S2, OA, DTX1, DTX2, and YTX retained 20%–50% better on the Strata-X and Bond Elut Plexa than on the HLB cartridge during the application and wash step. In addition, GYM, PTX2, AZA2, AZA3 were retained 10%–20% better on the Bond Elut Plexa than the Strata-X cartridge. Overall, the Bond Elut Plexa cartridge performed better than the other cartridges, and was therefore used for further optimization experiments.

The loading solvent strength, wash step, and elution step of the Bond Elut Plexa cartridge were optimized according to previous research on the extraction of LMTs in shellfish [41]. No breakthrough was observed when the crude clam methanol extract was diluted to 25% (*v*/*v*) methanol in water prior to application to the solid phase extraction (SPE) cartridge. At higher percentages of methanol (30%, 40%, 50% *v*/*v* methanol/water), breakthrough of OA, DTX1, DTX2, and YTX was observed. Thus, 25% *v*/*v* methanol was used as the loading solvent of the Bond Elut Plexa cartridge. Zero to sixty percent methanol mixtures with increments of 10% were applied to optimize the organic solvent strength of the wash step. OA, DTX1, DTX2, and YTX started to elute when wash solutions with 30% methanol were used. AZAs were retained on the cartridge with up to 50% methanol and PTX2 and SPX1 did not elute, even with 60% methanol. Therefore, a wash step of 3 mL 20% *v*/*v* methanol/water was incorporated to avoid losses during washing. In addition, no significant differences in the recovery of LMTs were obtained among the acidic, neutral, and alkaline wash solvent. Thus, a neutral wash step of 20% *v*/*v* methanol/water was incorporated in the final method. The elution step was performed with methanol. However, the recovery of YTX was somewhat lower (62.3%). According to previous research, YTX had a higher recovery when alkaline wash solutions were used [36]. In this study, when using 0.5 *v*/*v* ammonia solution to methanol, the recovery of YTX was increased to approximately 85%, without affecting the recoveries of the other LMTs. The optimized SPE purified method is described in Section 3.3.

**Figure 3:**
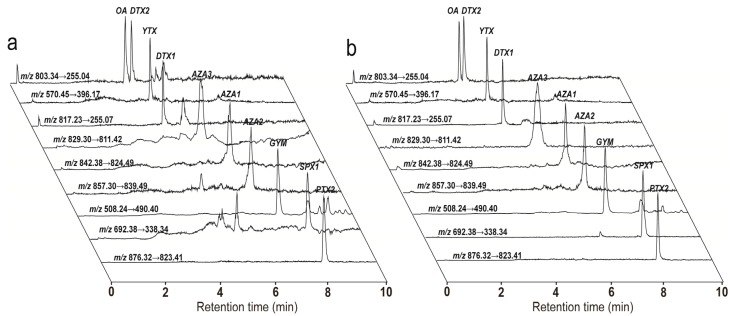
Chromatograms of a blank clam extract (*R. philippinarum*) spiked with ten LMT standards before (**a**); and after (**b**) the Bond Elut Plexa Cartridges SPE extraction.

**Figure 4:**
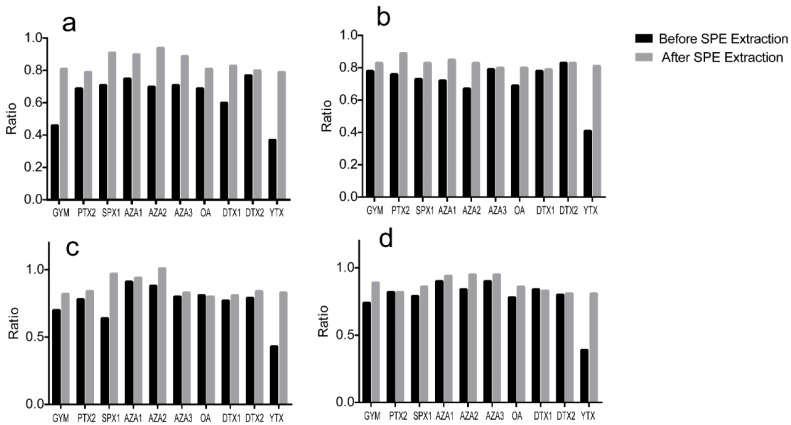
Matrix effect comparison of four blank clam matrices ((**a**) *M. meretrix*; (**b**) *R. philippinarum*; (**c**) *C. sinensis*; and (**d**) *M. veneriformis*) spiked with ten LMT standards before and after the Bond Elut Plexa Cartridges SPE extraction. Calibration curve slope ratios (*slope matrix*/*slope solvent*) were calculated to determine the matrix effects, slope values other than one denote ionization suppression (<1) or enhancement (>1).

As shown in Figure 3 and Figure 4, and Tables S1 and S2, the SPE cleanup procedure using the Bond Elut Plexa cartridges worked well for the 10 analytes by lowering the matrix effect and yielding satisfactory to excellent recoveries. Slope ratios for the 10 analytes varied from 0.39 to 0.91 in the four clam matrices before SPE cleanup, and then improved from 0.79 to 1.01 after the SPE extraction, which the matrix effect of clam matrices was no longer significant for LMT analysis. The average recovery of all toxins and matrices combined was acceptable (85.3%), as determined using the current UFLC gradient. For individual toxins, the recovery varied between 76.7% for YTX in *M. meretrix* extract and 100.0% for OA in *R. philippinarum* extract, and the relative standard deviation (RSD) values were all lower than 8.44%. Following the EU guideline (Document SANCO 10684/2009) (recovery in 70%–110%, RSD ≤ 20%) [48], the proposed SPE method was found to be accurate, with satisfactory recoveries for clam samples for *M. veneriformis*, *R. philippinarum*, *M. meretrix*, and *C. sinensis*.

### 2.2. Method Performance

The proposed UFLC-MS/MS method was validated by determining the specificity, matrix effect, accuracy (expressed as recovery), precisions, linearity, limit of detection (LOD), and limit of quantification (LOQ).

To establish the specificity of the method, blank samples of *M. veneriformis*, *R. philippinarum*, *M. meretrix*, and *C. sinensis* (five per species) were spiked with the 10 toxins, and then compared to the five non-spiked blank samples of each species. No significant interferences were observed at the retention time for all transitions and the target compounds were only present in the spiked clam samples at specific retention times, which suggests the high specificity of this method (Figure S3). Calibration curve slope ratios (matrix matched to pure solvent) were calculated to determine the matrix effects resulting in signal enhancement or suppression. As shown in Table 2, matrix effects were observed when the slope ratio values of the matrix curve of four clam tissue matrices were between 0.79 and 1.01, which indicated that the ionization suppression or signal enhancement did exist but was not significant [48]. All calibration curves exhibited good linear regression according to Table 2, with determination coefficients (*R*^2^) ranging from 0.9932 to 0.9996. Calibration ranges adequately covered variations in the amount of analyte in each sample. The LOQ and LOD corresponded to the lowest fortification level analyzed from the different matrices. As shown in Table 2, the LODs of 10 LMTs were not more than 0.11 μg/kg, whereas their LOQs were lower than 0.34 μg/kg. In general, these limits are considered acceptable to analyze LMTs in real clam samples. Precision studies were carried out for both intra-day and inter-day repeatability and reproducibility (see Tables S3 and S4). Matrix-matched calibration standards were prepared on each validation day. The results showed that the RSD of ten LMTs was between 0.89% and 5.06% for unhydrolyzed samples, whereas the RSD of OA, DTX1 and DTX2 was between 2.31% and 6.55% for hydrolysed samples, which were all within the acceptable range. The accuracy of the method was verified by measuring the recoveries from spiked blank samples of the four different matrices investigated at five replicates. As shown in Tables S3 and S4, the overall recovery rate of ten LMTs in unhydrolyzed samples was between 76.7% and 100.0%, and the RSD values were lower than 8.44%; whereas the recovery rate of OA, DTX1 and DTX2 was between 71.9% and 90.6% after hydrolysis, and the RSD values were lower than 10.95%. Following the EU guideline [48] (recovery in 70%–110%, RSD ≤ 20%), the proposed method was found to be accurate, with satisfactory recoveries for clam matrices cultivated in Jiangsu.

**Table 2:** Regression equation, correlation coefficients (*R*^2^), limit of detection (LOD) and quantification (LOQ), and matrix effect of the investigated LMTs.

Toxins	Solvent				*M. meretrix*					*R. philippinarum*					*C. sinensis*					*M. meretrix*				
	Curve	*R*^2^	LOD (μg/kg)	LOQ (μg/kg)	Curve	*R*^2^	LOD (μg/kg)	LOQ (μg/kg)	Ratio ^a^	Curve	*R*^2^	LOD (μg/kg)	LOQ (μg/kg)	Ratio ^a^	Curve	*R*^2^	LOD (μg/kg)	LOQ (μg/kg)	Ratio ^a^	Curve	*R*^2^	LOD (μg/kg)	LOQ (μg/kg)	Ratio ^a^
GYM	*y* = 122254*x* + 33042	0.9969	0.01	0.02	*y* = 99098*x* + 5125	0.9959	0.02	0.05	0.81	*y* = 101305*x* + 24625	0.9987	0.03	0.10	0.83	*y* = 99840*x* + 52125	0.9946	0.01	0.02	0.82	*y* = 108656*x* − 24458	0.9935	0.01	0.03	0.89
PTX2	*y* = 33852*x* + 27875	0.9943	0.01	0.03	*y* = 26711*x* + 10125	0.9975	0.10	0.34	0.79	*y* = 30143*x* − 8254.2	0.999	0.04	0.15	0.89	*y* = 28520*x* + 26017	0.9955	0.03	0.10	0.84	*y* = 27772*x* + 38250	0.9949	0.07	0.21	0.82
SPX1	*y* = 34501*x* + 13083	0.9982	0.01	0.03	*y* = 31367*x* + 139542	0.9974	0.02	0.06	0.91	*y* = 28724*x* + 139250	0.9936	0.23	0.76	0.83	*y* = 33315*x* + 66542	0.9981	0.01	0.04	0.97	*y* = 29504*x* + 188000	0.9983	0.01	0.05	0.86
AZA1	*y* = 163976*x* + 18500	0.9946	0.01	0.02	*y* = 147377*x* + 37208	0.9946	0.02	0.08	0.90	*y* = 138922*x* + 241000	0.9961	0.02	0.07	0.85	*y* = 153887*x* + 57458	0.995	0.04	0.12	0.94	*y* = 154834*x* + 8333.3	0.9938	0.04	0.12	0.94
AZA2	*y* = 41999*x* + 4795.8	0.9948	0.01	0.02	*y* = 39548*x* + 17808	0.9957	0.03	0.10	0.94	*y* = 34863*x* + 20754	0.9956	0.03	0.12	0.83	*y* = 42323*x* + 13600	0.9963	0.03	0.09	1.01	*y* = 39863*x* − 270.83	0.9932	0.02	0.07	0.95
AZA3	*y* = 52647*x* + 5875	0.9959	0.01	0.04	*y* = 47014*x* + 38983	0.9944	0.06	0.19	0.89	*y* = 42359*x* + 9708.3	0.9996	0.03	0.11	0.80	*y* = 43498*x* − 2700	0.9964	0.06	0.20	0.83	*y* = 49917*x* + 31592	0.9952	0.04	0.14	0.95
OA	*y* = 21901*x* + 5875	0.9959	0.02	0.08	*y* = 17703*x* + 9116.7	0.9974	0.03	0.10	0.81	*y* = 17482*x* − 1487.5	0.9994	0.02	0.07	0.80	*y* = 17613*x* − 2658.3	0.9994	0.03	0.11	0.80	*y* = 18871*x* − 11983	0.9988	0.02	0.06	0.86
DTX1	*y* = 40048*x* + 1545.8	0.9944	0.03	0.12	*y* = 33291*x* + 6500	0.997	0.04	0.12	0.83	*y* = 31723*x* − 1595.8	0.9971	0.02	0.08	0.79	*y* = 32429*x* −1529.2	0.9931	0.08	0.27	0.81	*y* = 33216*x* − 900	0.9964	0.04	0.12	0.83
DTX2	*y* = 9072.9*x* + 1250	0.9992	0.01	0.02	*y* = 7286.3*x* + 10404	0.9974	0.11	0.34	0.80	*y* = 7503.6*x* + 9516.7	0.9978	0.03	0.10	0.83	*y* = 7656.1*x* + 14129	0.9978	0.04	0.14	0.84	*y* = 7305.8*x* + 13629	0.9978	0.06	0.21	0.81
YTX	*y* = 10246*x* − 8650	0.9974	0.02	0.07	*y* = 8110.7*x* − 3329.2	0.9977	0.01	0.04	0.79	*y* = 8262*x* − 8270.8	0.9981	0.06	0.20	0.81	*y* = 8552.1*x* − 9491.7	0.9974	0.02	0.06	0.83	*y* = 8303.7*x* + 4625	0.9987	0.03	0.09	0.81

^a^ Matrix effects are calculated by slope of matrix curve/slope of solvent curve.

### 2.3. Occurrence of LMTs in Clams Cultivated in Jiangsu

**Figure 5:**
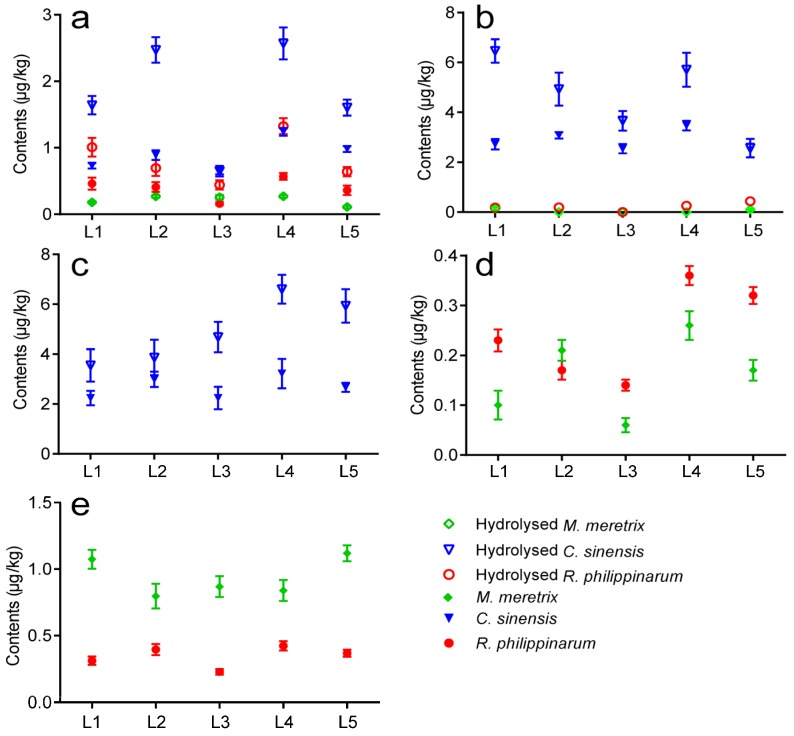
Distribution of LMTs in clams sampled at the five aquaculture zones along the coastline of Jiangsu province on May, 2014. (**a**) OA; (**b**) DTX1; (**c**) DTX2; (**d**) GYM; and (**e**) PTX2; L1: Maojia harbor; L2: Lvsi harbor; L3: Dafeng harbor; L4: Ganyu; and L5: Lianyun harbor. Error bars of triplicate analysis are included. Content scales are different for each toxins.

The *M. veneriformis*, *R. philippinarum*, *M. meretrix*, and *C. sinensis* samples collected from five sampling locations (Figure 1) along the coastline of Jiangsu province in May 2014 were pretreated three times and then examined with the validated method. The distribution of LMTs (μg/kg) in four clam species from five locations (Figure 5) was plotted based on the analytical results. As shown in Figure 5, toxin distribution varied among clam species: GYM and PTX2 were found in *R. philippinarum* and *M. meretrix* samples in all locations, free OA was observed in all *R. philippinarum* and *C. sinensis* samples, free DTX1 and DTX2 were detected in all *C. sinensis* samples, the esters of OA and DTX1 were detected in hydrolysed *R. philippinarum*, *C. sinensis* and *M. meretrix* samples, whereas the esters of DTX2 were only observed in hydrolysed *C. sinensis* samples. No AZAs, YTX, or SPX were detected in any sample.

Toxin concentrations varied with sampling locations (Figure 5). The highest concentrations of GYM, OA (including esters), and DTX1 (including esters) were all detected in Ganyu harbor, with GYM 0.36 μg/kg in *R. philippinarum*, OA 2.57 μg/kg, and DTX1 6.46 μg/kg in hydrolysed *C. sinensis*, whereas the highest level of PTX2 and DTX2 (including esters) were observed in Maojia harbor, with PTX2 1.11 μg/kg in *M. meretrix* and DTX2 6.60 μg/kg in hydrolysed *C. sinensis*. The lowest concentrations of GYM (0.06 μg/kg in *M. meretrix*), PTX2 (0.23 μg/kg in *R. philippinarum*), DTX2 and its esters (undetected in hydrolysed *R. philippinarum* and *M. meretrix*) were all observed in Dafeng harbor, whereas the lowest concentration of OA and its esters (0.11 μg/kg in hydrolysed *M. meretrix*), DTX2 and its esters (2.24 μg/kg in hydrolysed *C. sinensis*) were observed in Lianyun harbor.

The presence of OA and its analogues DTXs was first detected in shellfish on the Bohai coast (Xingcheng, Zhimao Bay) in China in 1999, in shellfish from Shanghai in 2005, in shellfish on the East China Sea coast (Ningbo and Ningde) in 2011, and later in shellfish collected from Qingdao in 2012 [29,36,49,50]. GYM was first confirmed in China in 2007 in oyster (*Dendostrea crenulifrea*) from Guangxi Beihai [51], whereas PTX2 and its analogues were observed for the first time in 2009 in oyster (*Crassostrea gigas*) originating from Dalian of the North Yellow Sea coast [33]. This study is the first report of OA, DTX1–2, PTX2, and GYM in clams cultivated in the aquaculture zones of Jiangsu Province, and their contents were much lower than the EU regulatory limits in shellfish flesh (see Tables S5) [47,52,53]. However, as some standards were not available to us (including the some PTX and YTX derivatives), the content of LMTs might be underestimated. Therefore, the potential risk to local consumers is still worth noting.

AZA1 was first discovered in 2010 in shellfish samples collected from Dalian and Guangzhou [54], later YTX and its analogues were reported in oysters collected from the coast of South China Sea [33], recently SPX was discovered in shellfish products located in Guangzhou [34]. Those toxins might become a new problem and pose a potential threat to consumers in China. However, in this study, none of the aforementioned toxins was detected in the clams cultivated along the coast of Jiangsu. As far as we know, the toxic microalgae that produce aforementioned toxins have not been found in this area yet, so this might be the reason we cannot detect AZA1-3, YTX and SPX1 in our collected clam samples. Further validation studies of toxic algae distribution in this area is needed.

### 2.4. Monthly Variations of LMTs in Clams Cultivated in Jiangsu during 2014–2015

**Figure 6:**
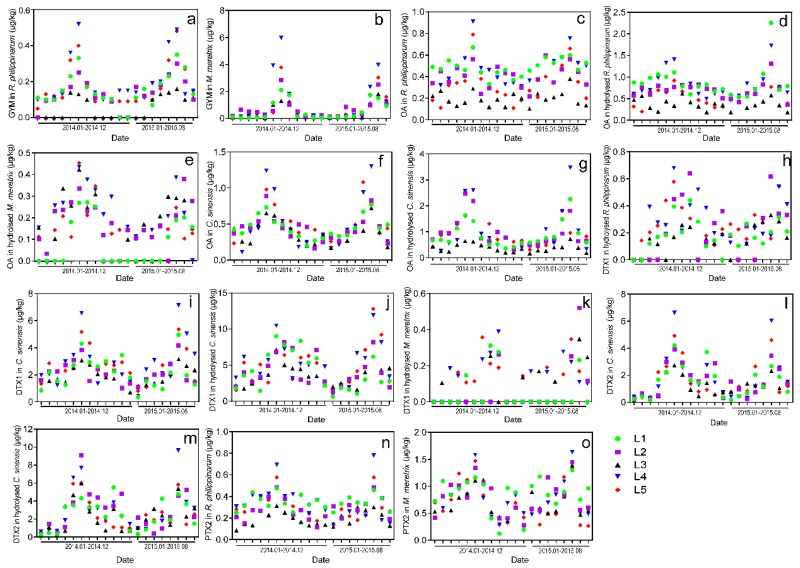
Monthly variations of LMTs (μg/kg) in the clam samples from the five sampling locations along the coastline of Jiangsu province from January 2014 to August 2015, (**a**,**b**) GYM content in *R. philippinarum* and *M. meretrix* samples; (**c**~**g**) OA content in *R. philippinarum*, *M. meretrix*, *C. sinensis* and their hydrolysed samples; (**h**~**k**) DTX1 content in hydrolysed *R. philippinarum, C. sinensis*, hydrolysed *C. sinensis* and *M. meretrix* samples; (**l**,**m**) DTX2 content in *C. sinensis* and its hydrolysed samples; (**n**,**o**) PTX2 content in *R. philippinarum* and *M. meretrix* samples; L1: Maojia harbor; L2: Lvsi harbor; L3: Dafeng harbor; L4: Ganyu; and L5: Lianyun harbor. Content scales are different for each graph.

Over 400 *M. veneriformis*, *R. philippinarum*, *M. meretrix*, and *C. sinensis* samples collected from five sampling locations along the coastline of Jiangsu province during the course of January 2014 to August 2015 were analyzed and pretreated under optimized analytical conditions. Same as previous results, GYM and PTX2 were present almost every month in *R. philippinarum* and *M. meretrix* samples, free OA was observed monthly in all *R. philippinarum* and *C. sinensis* samples, DTX1 and DTX2 were present almost every month in *C. sinensis* samples. No AZA1-3, YTX and SPX1 was detected in any sample, any month. The monthly variations of GYM, PTX2, OA/DTXs and their esterfied derivatives (μg/kg) in clam samples in each sampling location (Figure 6, Figure S4) were plotted according to the determination results.

As shown in Figure 6a,b, the concentrations of GYM in *M. meretrix* and *R. philippinarum* did not significantly change over the months, except during May through August for both year of sampling when sharp increases (in May) and sudden decreases (in August) occurred. The peak values of GYM were observed in July 2014 (*p* < 0.05, with the highest value at 5.96 μg/kg in L4) and July 2015 (*p* < 0.05, with the highest value at 3.97 μg/kg in L4) in *M. meretrix* samples, and in June 2014 (*p* < 0.05, with the highest value at 0.52 μg/kg in L4) and June 2015 (*p* < 0.05, with the highest value at 0.49 μg/kg in L4) in *R. philippinarum* samples from all five sampling locations. Moreover, the concentrations of GYM in *M. meretrix* samples (with the values varying from 0.05 to 5.96 μg/kg) were all higher than those of *R. philippinarum* (with the values varying from 0 to 0.52 μg/kg), which indicated that *M. meretrix* accumulated higher levels of GYM than *R. philippinarum*.

Similar patterns of seasonal variation were seen with OA and its esters. As shown in Figure 6c–g, the concentration of free OA and its esters sharply increased in May or June before rapidly dropping in July or August in all five locations during 2014–2015, except for the *R. philippinarum* samples collected from L1 and L3 and its hydrolysed samples collected from L3, which did not significantly change over the months. The peak values of OA and its esters were observed in June 2014 (*p* < 0.05, with the highest value at 1.40 μg/kg in L4) and June 2015 (*p* < 0.05, with the highest value at 2.26 μg/kg in L4) in hydrolysed *R. philippinarum* samples; in June (or May) 2014 (*p* < 0.05, with the highest value at 2.60 μg/kg in L4) and June (or May) 2015 (*p* < 0.05, with the highest value at 3.47 μg/kg in L4) in hydrolysed *C. sinensis* samples, and in June 2014 (*p* < 0.05, with the highest value at 0.44 μg/kg in L4) and June (or May) 2015 (*p* < 0.05, with the highest value at 0.38 μg/kg in L4) in hydrolysed *M. meretrix* samples from all sampling locations.

The seasonal variation patterns of DTXs and their esters in clam samples during 2014–2015 were different from those of GYM and OA. As shown in Figure 6h–m, sharp increases of DTX1&2 and their esters were observed in May and June followed by an abrupt decrease that began in July and continued until the nadir was reached in August or September in most of the clam samples. The concentrations increased again in October through November, before dropping in December. Peak values of DTX1 and its esterfied derivatives were reached in June 2014 (*p* < 0.05, with the highest value at 10.48 μg/kg in L4) and June 2015 (*p* < 0.05, with the highest value at 12.84 μg/kg in L5) in hydrolysed *C. sinensis* samples, the peak values of esterfied DTX1 were also reached in June 2014 (*p* < 0.05, with the highest value at 0.68 μg/kg in L5) and June 2015 (*p* < 0.05, with the highest value at 0.62 μg/kg in L2) in hydrolysed *R. philippinarum* samples, whereas the highest level of esterfied DTX1 was reached in July 2014 (*p* < 0.05, with the highest value at 0.36 μg/kg in L5) and June 2015 (*p* < 0.05, with the highest value at 0.35 μg/kg in L2). Meanwhile, the peak values of DTX2 and its esters were observed in June 2014 (*p* < 0.05, with the highest value at 9.11 μg/kg in L2) and June 2015 (*p* < 0.05, with the highest value at 9.65 μg/kg in L4) in hydrolysed *C. sinensis* samples from all five sampling locations.

PTX2 variations in *M. meretrix* and *R. philippinarum* samples were similar to those of DTX1. As shown in Figure 6n,o, the concentrations of PTX2 significantly increased during April and June, rapidly decreased during July and August, reaching the lowest level in September, before increasing again from October to November, with a final decrease in December. Peak values of PTX2 were reached in June 2014 (*p* < 0.05, with the highest value at 6.55 μg/kg in L4) and June 2015 (*p* < 0.05, with the highest value at 7.15 μg/kg in L4) in *C. sinensis* samples.

Additionally, we found that the contents of detected LMTs varied with different aquaculture locations. As shown in Figure S4, Most of the peak levels of GYM, OA, DTXs, and PTX2 were observed in the clam samples collected in L4 (Ganyu). The average contents of GYM, DTX1 (including esters), DTX2 (including esters), OA esters in hydrolysed *M. meretrix* were markedly higher (*p* < 0.05) in clam samples in L4, whereas the contents of PTX2 and OA were significantly higher (*p* < 0.05) in the samples cultivated in L1 (Maojia harbor). L3 (Dafeng harbor) had the lowest average content of LMTs in clam samples compared to the other regions.

LMTs are secondary metabolites produced by marine toxic algae that accumulate in bivalves via feeding behavior. Previous studies discovered that toxic algae was primarily found in the coastal waters of Qingdao (Yellow sea, Shandong province) from May to August, and the contents of LMTs (including OA, DTX1, and PTX2) were pronouncedly increased during June through August [55]. High levels of LMTs were subsequently detected in coastal seawater during this period [31]. Similarly, in this study, we were the first to discover that the LMTs (including GYM, PTX2, DTX1, DTX2, and OA) all reached their peak levels during May to August in clam samples harvested in Jiangsu during the 2014 and 2015 seasons. As the coastal area of Jiangsu is a major culturing area for clams, the results of this study can (1) provide a specific and sensitive monitoring strategy to track the occurrence of LMTs in predominant clam samples cultivated in Jiangsu and to assess risk in order to ensure the food safety for the local consumers; and (2) help local farmers, consumers, researchers, and producers of medicinal preparations select the proper season and locations in which to collect clams in this area.

Although the occurrence and seasonal variations of LMTs in clam samples harvested in Jiangsu have been elucidated in this study, the distribution pattern and population dynamics of the source of LMTs—toxic algae—in the coastal water of this area remain unclear to date. Moreover, the transfer mechanism of LMTs from algae to clams as well as their absorption and metabolism pathways in different clam species are also not well understood. Thus, further studies on the seasonal variations of algae cell densities in seawater and the toxin production status of toxic algae species in Jiangsu province are needed. Future research on the transfer mechanism of LMTs to clams as well as toxin metabolism pathways in different clam species is also strongly recommended.

## 3. Experimental Section

### 3.1. Standards and Reagents

Okadaic acid (OA), yessotoxin (YTX), azaspiracids (AZA1, AZA2, and AZA3), pectenotoxin-2 (PTX2), gymnodimine (GYM), dinophysistoxins (DTX1, DTX2), and 13-desmethylspirolide (SPX1) were all purchased from the National Research Council, Institute for Marine Biosciences (NRC-CNRC), Halifax, NS, Canada.

MS-grade acetonitrile and HPLC-grade methanol were obtained from Merck, Darmstadt, Germany. Ammonium hydroxide (28%–30%) was purchased from Tedia, Fairfield, CA, USA. Ultra-pure water was generated using the Milli-Q system (Millipore, Billerica, MA, USA). All other chemicals and reagents were of analytical grade.

### 3.2. Sample Collections

From January 2014 to August 2015, our group collected the dominant clam species (*M. veneriformis*, *R. philippinarum*, *M. meretrix*, and *C. sinensis*) monthly along the coast of Jiangsu, China. As shown in Figure 1, the specific culturing sites were Maojia harbor (L1, 121°40ʹ E, 32°01ʹ N), Lvsi harbor (L2, 121°37ʹ E, 32°05ʹ N), Dafeng harbor (L3, 120°50ʹ E, 33°16ʹ N), Ganyu (L4, 121°49ʹ E, 34°29ʹ N), and Lianyun harbor (L5, 119°20ʹ E, 34°46ʹ N). After collection, samples were starved in an aquarium for 24 h to evacuate gut contents. Flesh was then excavated from the shells and stored at −20 °C. All collected samples were authenticated by an expert and voucher specimens were deposited at the College of Pharmacy, Nanjing University of Chinese Medicine, Nanjing, China.

### 3.3. Standards and Extracts Preparations

A stock solution containing 313 ng/mL of GYM, 441 ng/mL of PTX2, 701 ng/mL of SPX1, 310 ng/mL of AZA1, 254 ng/mL of AZA2, 260 ng/mL of AZA3, 312.5 ng/mL of OA, 758 ng/mL of DTX-1, 781 ng/mL of DTX-2, and 280 ng/mL of YTX was prepared in methanol. This stock solution was further diluted with methanol to provide at least six different concentrations. The concentrations of calibration standards were ranging from as follows: GYM, 0.49–125.2 ng/mL; PTX2, 0.69–176.4 ng/mL; SPX1, 0.55–280.4 ng/mL; AZA1, 0.48–124 ng/mL; AZA2, 0.40–101.6 ng/mL; AZA3, 0.41–104 ng/mL; OA, 0.49–124 ng/mL; DTX1, 0.47–303.2 ng/mL; DTX2, 0.61–390.5 ng/mL and YTX, 1.75–112 ng/mL.

The clam LMTs extract was prepared using Gerssen’s method [41,42] as a reference. Briefly, the clam tissues were homogenized with an electric blender. Five grams of clam homogenate was extracted in triplicate with 25 mL methanol by ultrasonic assisted extraction. The extraction was performed for 30 min, and then the sample was centrifuged 10 min at 2000× *g*. The supernatants were combined and evaporated by a rotary evaporator (Buchi, R210, Flawil, Switzerland) until dry, with the water temperature at 40 °C to obtain the crude clam extract. The crude clam extract was diluted with 6 mL of 25% *v*/*v* methanol, then applied on the Bond Elut Plexa cartridge (60 mg, 3 mL, Agilent, Santa Clara, CA, USA). The cartridge was washed with 3 mL 20% *v*/*v* methanol to remove polar components. The LMTs were then eluted from the cartridge using 3 mL methanol containing 0.5% *v*/*v* ammonium hydroxide. The purified extracts were dried with nitrogen flow at 4 °C and re-suspended in 100 μL methanol, store overnight in −20 °C refrigerator, then centrifuged 5 min at 12,000 rpm in a refrigerated centrifuge (SIGMA, 3–18 K, Osterode, Germany), and, finally, the supernatant was diluted ten times with methanol before injected into the UFLC-MS/MS system (Shimadzu, Kyoto, Japan; Applied Biosystems, Foster City, CA, USA).

The blank clam samples (five samples per species) that had not been exposed to LMTs were provided by Professor Xi-he Wan, Institute of Oceanology and Marine Fisheries, Nantong, Jiangsu. The blank matrix extracts including *M. veneriformis*, *R. philippinarum*, *M. meretrix*, and *C. sinensis* were prepared in the same way as the clam LMT extract. Matrix matched calibration standards were prepared by the addition of different concentrations of standard solution to the blank clam extract. The matrix matched calibration standards were freshly prepared before use. 

In order to quantify the total content of OA and DTXs, an alkaline hydrolysis is required [47]. The hydrolysis extract was prepared mainly according to the EU-Harmonised standard operating procedure for determination of LMTs in molluscs by LC-MS/MS (Version 5) [47]. Briefly, the crude clam extract of Section 3.3 was diluted with 2 mL of methanol in a test tube, 250 μL of 2.5 M sodium hydroxide solution was added to the extract. The contents of the closed tube were mixed and the tube was placed in a water bath at 76 °C. After 45 min, the hydrolysed extract was cooled to room temperature and neutralized with 250 μL of 2.5 M hydrochloric acid, then mixed with 5.5 mL water, and purified by SPE according to Section 3.3. The purified extract was dried with nitrogen flow and re-suspended in 100 μL methanol, stored overnight at −20 °C, centrifuged and the supernatant was diluted ten times with methanol before analysis.

### 3.4. UFLC-MS/MS Method

Chromatographic analysis was performed on a Prominence™ UFLC system (Shimadzu, Kyoto, Japan). The separation was achieved on an Xbridge™ C_18_ column (50 mm × 2.1 mm; i.d. 2.5 μm, Waters, Ireland) with a guard cartridge at a temperature of 35 °C. The mobile phase was composed of acetonitrile (A); and 5 mM ammonium hydroxide aqueous solution (B), with an elution gradient as follows: 0–10 min, 10%–90%; 10–12 min, 90% of A. The flow rate was set to 0.3 mL/min, and an injection volume of 2 μL was selected. All the toxins were eluted within 10 min. During the rest time, the column was cleaned, readjusted to the initial conditions, and equilibrated.

The triple-quadrupole linear ion trap mass spectrometers (5500 Q-Trap™, Applied Biosystems) equipped with a TurboIonSpray™ (Applied Biosystems) source were tested in both positive and negative ionization mode. Instrument control, data acquisition, and the processing were performed using the associate Analyst 1.5.2 software. MS/MS data acquisition was performed in the MRM mode. In order to obtain maximum sensitivity for detection of the LMTs, the ion source temperature (TEM) was set at 550 °C and the ion source voltages (IS) were set at 5.5 kV and −4.5 kV in positive and negative ion modes, respectively. Ion source gas 1 and 2 (GS 1 & 2) were set at 55 arbitrary units. Curtain gas (CUR) was 35 arbitrary units. The analyte specific parameters are all shown in Table 1.

### 3.5. Method Validation

Validation of methods was performed according to Document SANCO 10684/2009 [48]. The validation included the determination of accuracy (expressed as recovery), precisions, linearity, LOD, LOQ, and matrix effect.

Precision studies were carried out for both intra-day and inter-day repeatability and reproducibility, by running six blank *M. meretrix* matrix extracts spiked at three levels (low, intermediate, and high) of LMT standards on a single day, and reproducibility was quantified using the six replicate samples spiked at the same level on five different days. The accuracy of the method was verified by measuring the recoveries from spike the blank clam matrices of four species (five samples per species) with known amount of standard solutions. Five replicates were performed to determine the RSD, and the average recovery was estimated using the following formula: recovery (%) = (detection quantity/quantity added) × 100%.The linear calibration curves were obtained at six different concentrations for the toxins in pure solvent (methanol), whereas the matrix matched calibration curves were obtained at six different concentrations of LMTs in four blank clam matrices. The limit of quantification (LOQ) and the limit of detection (LOD) were analyzed by diluting the toxin standards in the pure solvent and different clam matrices to a series of concentrations with methanol then determined at a signal-to-noise (S/N) ratio of three and 10, respectively. Calibration curve slope ratios (matrix matched to solvent) were calculated to determine the matrix effects resulting in signal enhancement and suppression [56]. The slopes of the matrix matched calibration curves were compared with the slopes obtained from the pure solvent at the same concentration levels. A slope ratio (slope matrix/slope solvent) of one indicates that the matrix does not significantly suppress or enhance the response of the MS. Slope values other than one denote ionization suppression (<1) or enhancement (>1).

As no esterified standards of OA and DTXs were available, the performance of the alkaline hydrolysis was validated by subjecting OA, DTX1 and DTX2 to hydrolysis conditions. Briefly, precision tests were performed by using the blank samples spiked with different levels of OA, DTX1 and DTX2 and then prepared the same way as described in Section 3.4. Similarly, the accuracy tests were executed by spiking a known amount of standard solutions of OA, DTX1 and DTX2 to four blank clam matrices. The spiked samples were extracted, hydrolysed and purified according to Section 3.4. Then, the toxin recovery was calculated.

### 3.6. Statistical Analysis

All experiments were performed at least three times. Analyses of variance (ANOVA) were performed using the Student-Newman-Keuls (S-N-K) procedure in Statistical Package for the Social Sciences 22.0 (SPSS Inc., Chicago, IL, USA). If the statistical analysis (*F* test) showed a significant effect of treatment (harvest date, *p* < 0.05), Fisher’s least significant difference (LSD) was used to compare means.

## 4. Conclusions

In summary, this study demonstrated that:

(1) An offline SPE coupled with the UFLC-MS/MS method was developed for the first time to determine the presence of 10 LMTs, including GYM, AZA1-3, DTX1&2, OA, PTX2, SPX1, and YTX in *M. veneriformis*, *R. philippinarum*, *M. meretrix*, and *C. sinensis* samples. This method is validated by precision, sensitivity, repeatability, recovery, and suitability for the routine monitoring of typical LMTs in clam samples.
(2) The validated method was successfully applied to determine the presence of LMTs in clam samples collected from five aquaculture zones of Jiangsu Province. Results showed that GYM and PTX2 were found in *R. philippinarum* and *M. meretrix* samples, OA was observed in *R. philippinarum* and *C. sinensis* samples, and DTX1 and DTX2 were detected only in *C. sinensis* samples from all five locations in May 2014.
(3) The monthly variations of LMTs (including GYM, PTX2, DTX1, DTX2, and OA) in different clam species were verified from January 2014 to August 2015, and the highest concentrations of the aforementioned LMTs were observed during May through August. Although the toxin levels of all samples were much lower than the EU regulatory limits, they might be underestimated due to the restricted toxin variety we detected.
(4) The LMTs monitoring method we developed in this study could evaluate the potential risk and safety of clams for local consumers. The seasonal variation information we offered could guide local farmers or consumers to choose appropriate season or locations for clam harvesting. Further studies should focus on improving our current understanding of the distribution pattern and population dynamics of toxic algae in this area as well as the transportation and transformation of these toxins in the different clam species.

## Supplementary Materials

The following are available online at www.mdpi.com/2072-6651/8/1/8/s1.
