# Supplementary material for: Occurrence and Seasonal Variations of Lipophilic Marine Toxins in Commercial Clam Species along the Coast of Jiangsu, China
Source: Toxins (Basel). 2015 Dec 25;8(1):8. doi: 10.3390/toxins8010008 (PMC4728530; doi:10.3390/toxins8010008)
Supplement: Supplementary file 1 [file toxins-08-00008-s001.pdf]

Supplementary Materials: Occurrence and Seasonal Variations of Lipophilic Marine Toxins in Commercial Clam Species along the Coast of Jiangsu, China

Xin-Zhi Wang, Ying Cheng, Na Li, Hong-Mei Wen, Rui Liu, Chen-Xiao Shan, Chuan Chai and Hao Wu

Table S1. Recovery test of the ten investigated LMTs.

Toxins	*M. meretrix*		*R. philippinarum*		*C. sinensis*		*M. veneriformis*	
	Avg (n = 5)	RSD %	Avg (n = 5)	RSD %	Avg (n = 5)	RSD %	Avg (n = 5)	RSD %
GYM	81.2	6.45	80.4	6.88	79.7	8.44	89.5	7.01
SPX1	81.0	8.19	89.9	5.11	82.1	4.08	80.1	3.77
PTX	89.4	4.29	80.3	5.38	90.7	5.26	80.3	6.48
AZA1	87.7	3.01	82.3	7.61	90.5	2.06	80.5	4.07
AZA2	80.9	3.95	81.3	3.79	91.6	4.2	92.8	5.91
AZA3	90.2	4.22	86.5	4.17	85.2	3.84	93.9	8.05
OA	94.4	2.96	100.0	2.41	96.1	3.65	91.7	5.13
DTX1	85.1	1.93	83.9	4.25	91.3	4.37	84.2	3.53
DTX2	86.4	5.78	87.2	4.85	83.1	6.79	81.0	5.09
YTX	76.7	1.37	80.6	5.26	81.5	5.58	78.8	4.17

Table S2. Recovery test of OA, DTX1 and DTX2 after alkaline hydrolysis.

Toxins	*M. meretrix*		*R. philippinarum*		*C. sinensis*		*M. veneriformis*	
	Avg (n = 5)	RSD %	Avg (n = 5)	RSD %	Avg (n = 5)	RSD %	Avg (n = 5)	RSD %
OA	83.2	9.95	89.3	8.32	90.6	6.62	81.9	7.46
DTX1	75.2	6.12	84.3	7.51	78.0	5.37	72.3	6.13
DTX2	79.3	7.81	79.3	6.13	71.9	8.13	78.7	10.95

Table S3. Precision test of the ten investigated LMTs [a].

Compounds	Spiked Levels (μg/kg)	Intra-Day Precision (n = 6)		Inter-Day Precision (n = 5)
		Mean Value (μg/kg) ± SD	RSD %	RSD %
GYM	0.1	0.08 ± 0.00	0.89	1.29
	1.04	0.89 ± 0.02	1.9	2.04
	10.43	8.67 ± 0.27	3.16	3.14
SPX1	0.24	0.22 ± 0.01	2.91	3.04
	2.35	1.98 ± 0.07	3.3	3.35
	23.5	20.36 ± 0.77	3.77	4.89
PTX	0.15	0.14 ± 0.01	2.92	2.35
	1.47	1.28 ± 0.05	3.63	3.99
	14.69	13.12 ± 0.66	5.01	5.65
AZA1	0.1	0.09 ± 0.00	2.94	3.06
	1.03	0.98 ± 0.02	2.5	2.09
	10.33	9.03 ± 0.33	3.65	4.95
AZA2	0.13	0.11 ± 0.00	1.41	2.65
	1.27	1.12 ± 0.02	1.87	3.94
	12.7	11.36 ± 0.28	2.5	4.52

Table S3. *Cont.*

Compounds	Spiked Levels (μg/kg)	Intra-Day Precision (n = 6)		Inter-Day Precision (n = 5)
		Mean Value (μg/kg) ± SD	RSD %	RSD %
AZA3	0.13	0.12 ± 0.00	2.72	2.11
	1.3	1.19 ± 0.01	1.14	2.39
	13	12.01 ± 0.5	4.18	3.97
OA	0.1	0.09 ± 0.00	4.47	4.01
	0.98	0.98 ± 0.04	4.04	5.11
	10.42	10.12 ± 0.51	5.06	5.53
DTX1	0.15	0.14 ± 0.00	1.01	2.92
	1.52	1.39 ± 0.02	1.71	3.26
	15.15	14.06 ± 0.38	2.7	4.09
DTX2	0.16	0.15 ± 0.00	1.65	1.97
	1.56	1.46 ± 0.02	1.09	2.35
	15.62	13.05 ± 0.35	2.71	3.21
YTX	0.14	0.12 ± 0.01	4.58	5.07
	1.4	1.23 ± 0.03	2.79	2.59
	14	12.94 ± 0.47	3.67	4.01

[a] Precision test was conducted in *R. philippinarum* matrices.

Table S4. Precision test of OA, DTX1 and DTX2 after alkaline hydrolysis [a].

Compounds	Spiked Levels (μg/kg)	Intra-Day Precision (n = 6)		Inter-Day Precision (n = 5)
		Mean Value (μg/kg) ± SD	RSD %	RSD %
OA	0.10	0.09 ± 0.00	6.01	6.19
	1.96	1.61 ± 0.11	6.55	6.02
	19.60	16.57 ± 0.83	5.03	5.31
DTX1	0.15	0.14 ± 0.00	3.99	3.81
	3.03	2.60 ± 0.14	5.38	6.10
	30.31	25.87 ± 1.41	5.45	5.37
DTX2	0.16	0.15 ± 0.00	2.31	3.84
	3.12	2.82 ± 0.16	5.65	5.93
	31.24	28.38 ± 1.06	3.74	4.15

[a] Precision test was conducted in hydrolysed *R. philippinarum* matrices.

Table S5. Current permitted levels in EU legislation and levels proposed by EFSA [a].

Toxin	EU Legislation (μg/kg)	EFSA Opinion (μg/kg)
OA and DTXs	160 [b]	45
PTXs	-	120
YTXs	1000	3750
AZAs	160	32

[a] The European Food Safety Authority; [b] including PTXs.

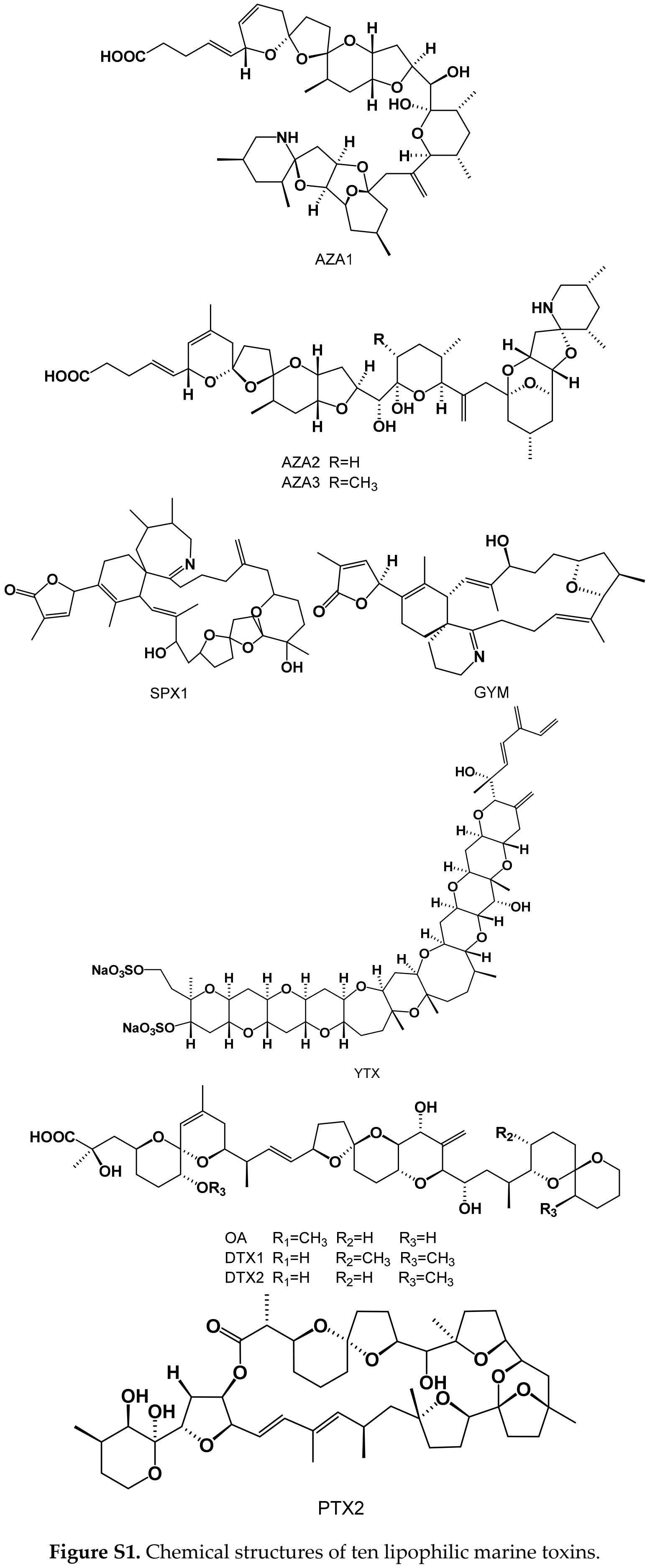

Figure S1. Chemical structures of ten lipophilic marine toxins.

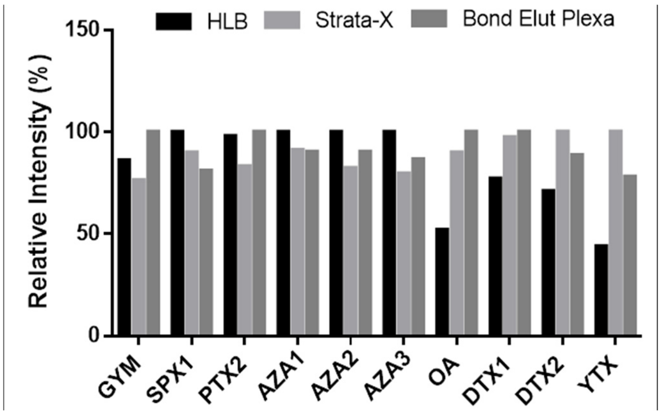

Figure S2. Relative intensity of LMTs on three different polymetric sorbents with the preliminary SPE protocol. Highest intensity per individual toxin is set at 100%. Wash with 25% (*v*/*v*) methanol and elution with methanol.

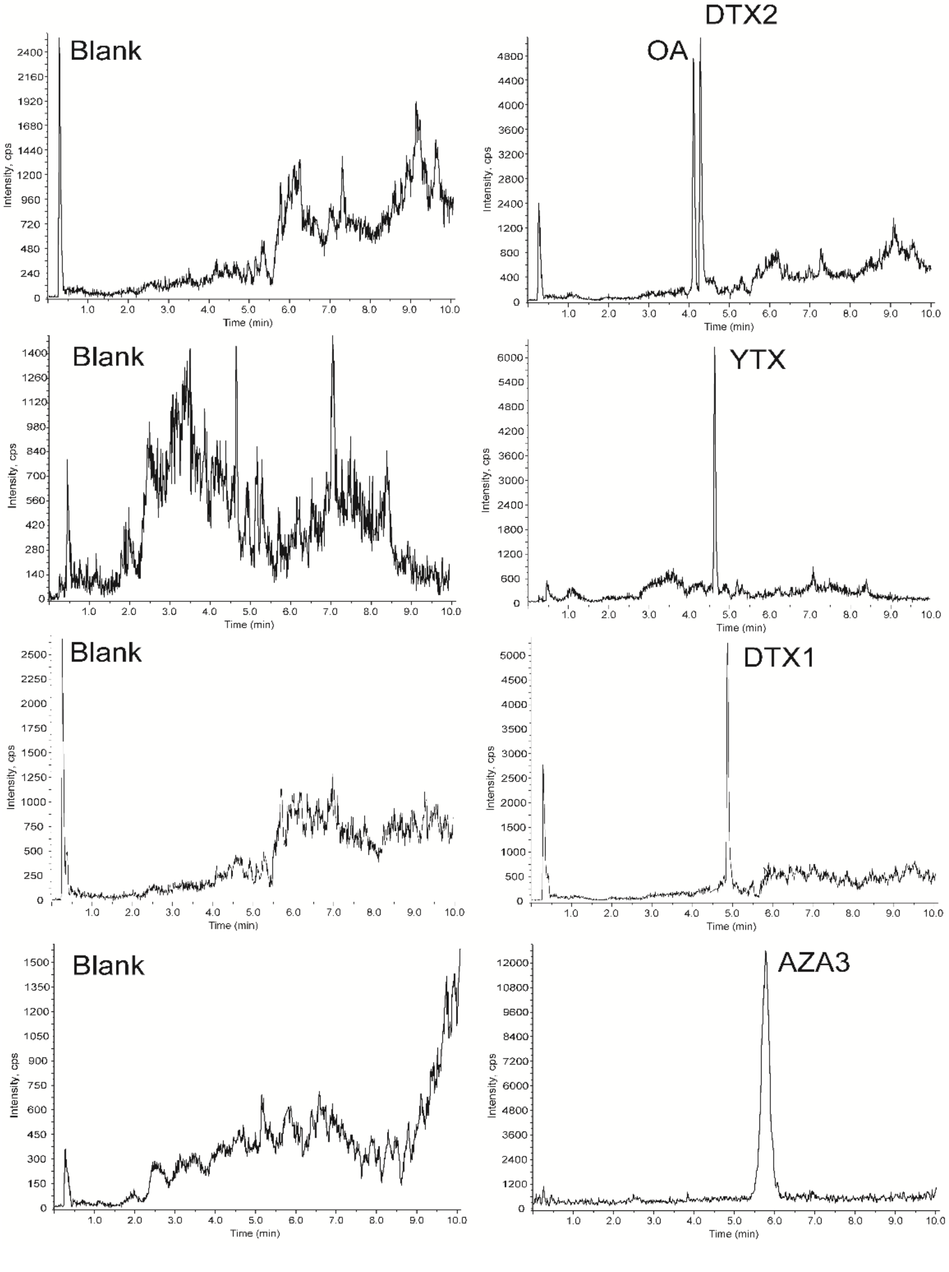

Figure S3. *Cont.*

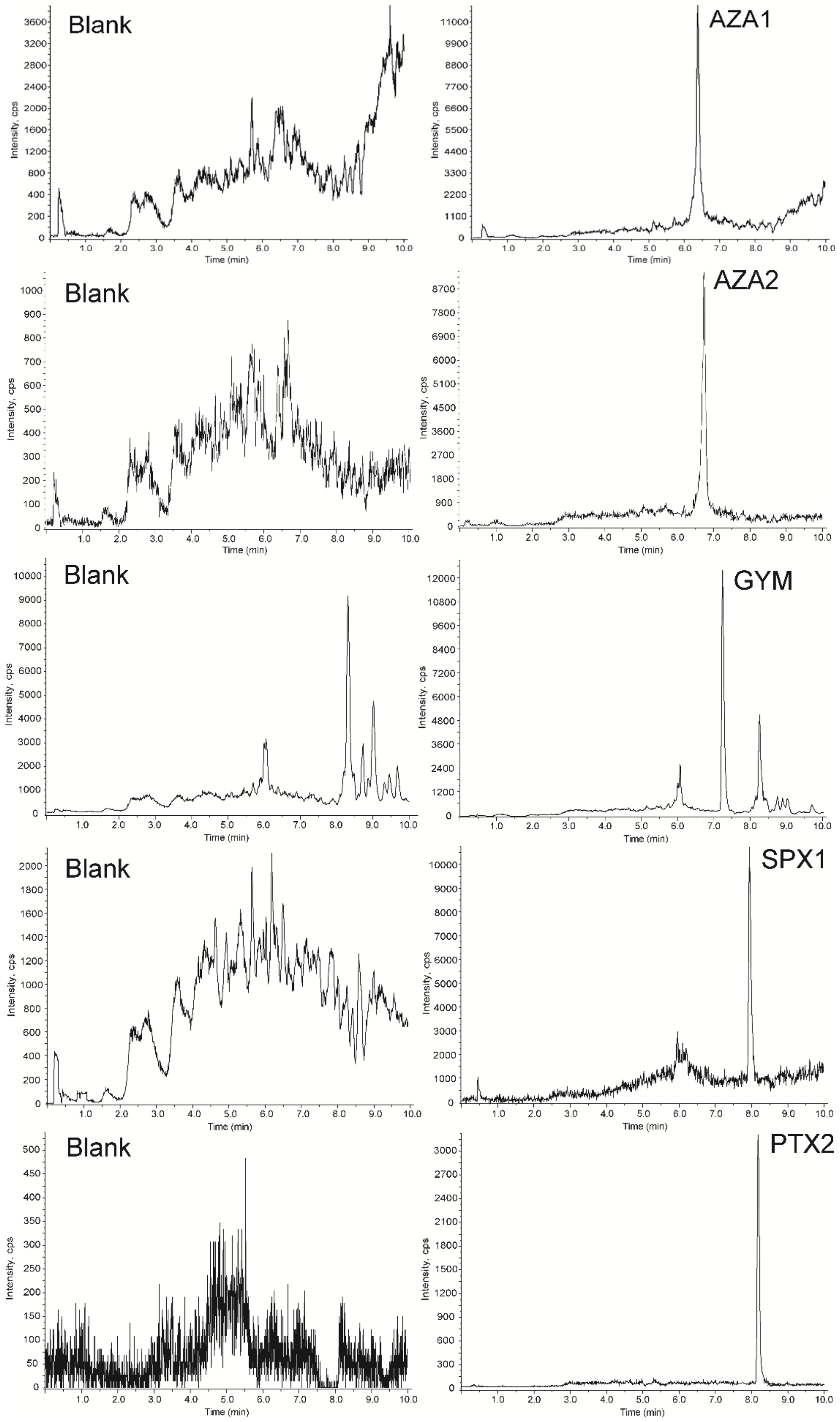

Figure S3. UFLC-TQ-MS/MS MRM chromatograms of the blank *M. meretrix* matrice and spiked samples. UFLC-TQ-MS/MS MRM chromatograms of the blank *M. meretrix* matrice and spiked samples. The chromatograms of blank samples are magnified to ensure there was no target compound peak exist.

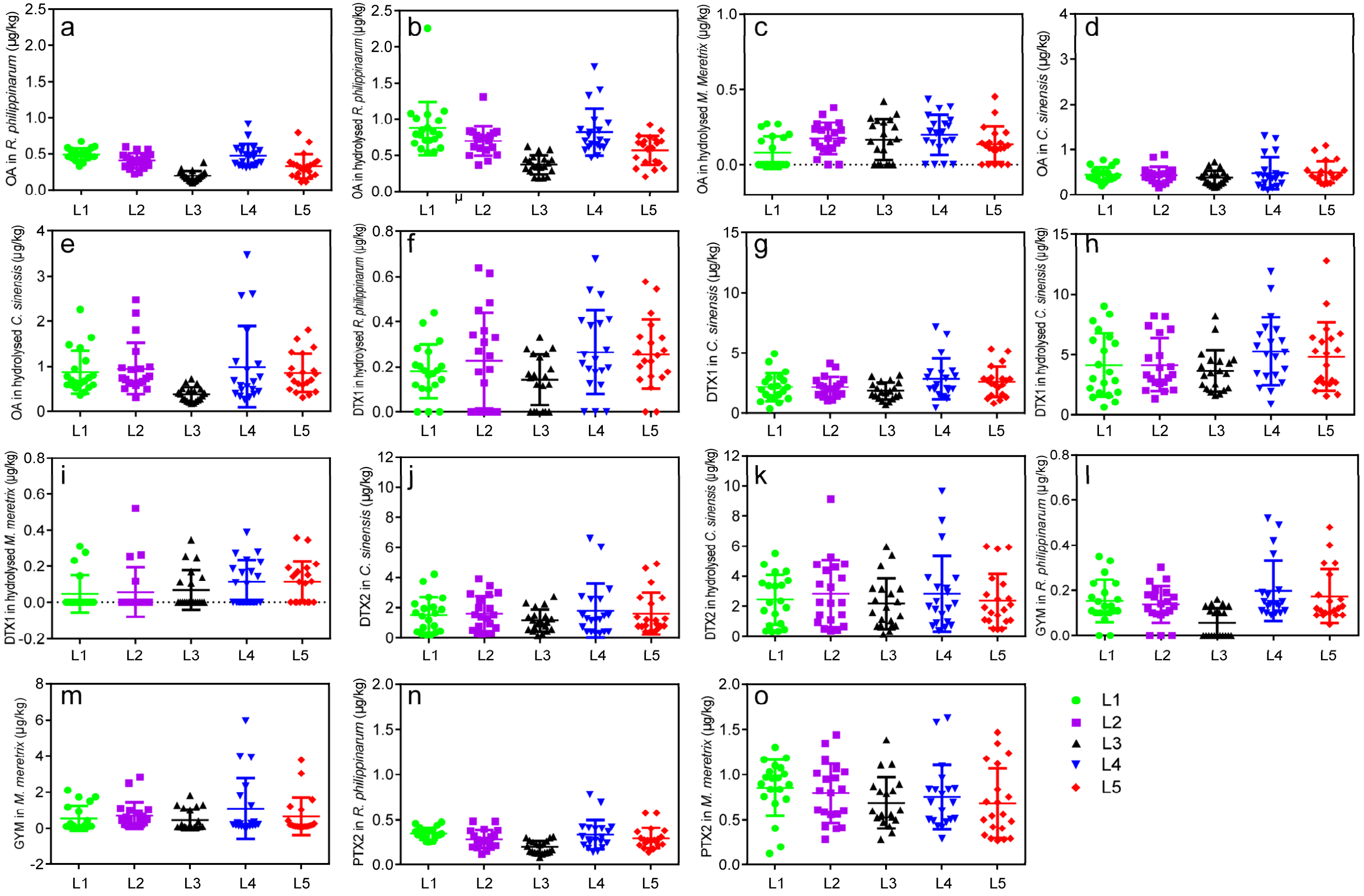

Figure S4. Distribution of LMTs in clam samples from different sampling locations in Jiangsu, China, during January 2014 to August 2015. (**a**)~(**e**) OA content in *R. philippinarum*, *M. meretrix*, *C. sinensis* and their hydrolysed samples, (**f**)~(**i**) DTX1 content in hydrolysed *R. philippinarum*, *C. sinensis*, hydrolysed *C. sinensis* and *M. meretrix* samples, (**j**,**k**) DTX2 content in *C. sinensis* and its hydrolysed samples, (**l**,**m**) GYM content in *R. philippinarum* and *M. meretrix* samples, (**n**)~(**o**) PTX2 content in *R. philippinarum* and *M. meretrix* samples; L1: Maojia harbor, L2: Lvsi harbor, L3: Dafeng harbor, L4: Ganyu, and L5: Lianyun harbor. Each dot represents one month across the sampling period grouped by sample location and content scales are different for each graph.
